# DT-diaphorase activity in NSCLC and SCLC cell lines: a role for fos/jun regulation

**DOI:** 10.1038/sj.bjc.6690268

**Published:** 1999-04

**Authors:** J K Kepa, D Ross

**Affiliations:** Department of Pharmaceutical Sciences, Molecular Toxicology and Environmental Health Sciences Program, School of Pharmacy and Cancer Center, University of Colorado Health Sciences Center, Denver, CO, 80262, USA

**Keywords:** NAD(P)H:quinone reductase, DT-diaphorase, fos/jun, NSCLC, SCLC

## Abstract

To assess the potential differential lung tumour expression of NAD(P)H:quinone reductase (NQO1), the human (h) NQO1 promoter was characterized in gene transfer studies. A deletion panel of 5′ flanking hNQO1 promoter constructs was made and tested in transient transfection assays in NSCLC and SCLC cell lines. The largest hNQO1 construct (–1539/+115) containing the antioxidant response element (ARE), exhibited robust levels of reporter activity in the NSCLC (H460, H520, and A549) cell lines and expression was over 12 to 77-fold higher than the minimal (–259/+115) promoter construct. In contrast, there was little difference in promoter activity between the largest and minimal promoter construct in the SCLC (H146, H82 and H187) cell lines. Deletion of the sites for NFκB and AP-2 and the XRE did not significantly affect hNQO1 promoter activity in either the NSCLC or SCLC cell lines. Robust promoter activity in NSCLC lines was mediated by a 359 bp segment of the proximal promoter that contained a canonical AP-1 binding site, TGACTCAG, within the ARE. Gel supershift assays with various specific Fos/Jun antibodies identified Fra1, Fra2 and Jun B binding activity in NSCLC cells to a promoter fragment (–477 to –438) spanning the AP-1 site, whereas SCLC do not appear to express functional Fra or Jun B. These results suggest a possible role for AP-1 activity in the differential expression of hNQO1 in NSCLC. © 1999 Cancer Research Campaign


*British Journal of Cancer* (1999) **79**(11/12), 1679–1684
© 1999 Cancer Research Campaign
Article no. bjoc.1998.0268

### DT-diaphorase activity in NSCLC and SCLC cell lines: a role for fos/jun regulation

**JK Kepa and D Ross**

Department of Pharmaceutical Sciences, Molecular Toxicology and Environmental Health Sciences Program, School of Pharmacy and Cancer Center, University of Colorado Health Sciences Center, Denver, CO 80262, USA

**Summary** To assess the potential differential lung tumour expression of NAD(P)H:quinone reductase (NQO1), the human (h) NQO1 promoter was characterized in gene transfer studies. A deletion panel of 5′ flanking hNQO1 promoter constructs was made and tested in transient transfection assays in NSCLC and SCLC cell lines. The largest hNQO1 construct (–1539/+115) containing the antioxidant response element (ARE), exhibited robust levels of reporter activity in the NSCLC (H460, H520, and A549) cell lines and expression was over 12 to 77-fold higher than the minimal (–259/+115) promoter construct. In contrast, there was little difference in promoter activity between the largest and minimal promoter construct in the SCLC (H146, H82 and H187) cell lines. Deletion of the sites for NFκB and AP-2 and the XRE did not significantly affect hNQO1 promoter activity in either the NSCLC or SCLC cell lines. Robust promoter activity in NSCLC lines was mediated by a 359 bp segment of the proximal promoter that contained a canonical AP-1 binding site, TGACTCAG, within the ARE. Gel supershift assays with various specific Fos/Jun antibodies identified Fra1, Fra2 and Jun B binding activity in NSCLC cells to a promoter fragment (–477 to –438) spanning the AP-1 site, whereas SCLC do not appear to express functional Fra or Jun B. These results suggest a possible role for AP-1 activity in the differential expression of hNQO1 in NSCLC.

**Keywords:** NAD(P)H:quinone reductase; DT-diaphorase; fos/jun; NSCLC; SCLC

Many human tumors have elevated DT-diaphorase activities, such as liver (Beyer et al, 1987), colon (Schlager et al, 1990), lung (Malkinson et al, 1992) and breast (Berger et al, 1985). In the case of lung tumours, elevated NQO1 activity has recently been reported in non-small cell lung cancer (NSCLC) cell lines and primary tumours relative to SCLC and uninvolved lung (Schlager and Powis, 1990; Malkinson et al, 1992; Smitskamp-Wilms et al, 1995). This work validated NSCLC as a potential target against which anti-tumour drugs, which are efficiently bioactivated by NQO1, can be tested for their cytotoxicity. Biochemical studies have already demonstrated that NQO1 activity is induced by a wide range of chemicals including polycyclic aromatic hydrocarbons, azo dyes and phenolic antioxidants (Benson et al, 1980; DeLong et al, 1986; Joseph et al, 1994; Talalay and Prochaska, 1987; Talalay et al, 1988). Sequence analysis of the 5′ flanking region of the human NQO1 gene shows the presence of an activator protein 2 (AP-2), antioxidant response element (ARE), also called the electrophile response element (EpRE), a xenobiotic response element (XRE) and a nuclear factor κB (NFκB)-like element within 900 bp of the transcriptional start site (Jaiswal, 1991). Interestingly, the ARE in the human NQO1 gene (–477 to –438) actually contains a centrally-located perfect AP-1 site, flanked by two imperfect AP-1 elements. In this study, we examined the mechanisms underlying the differential basal expression of hNQO1 in NSCLC versus SCLC. Initially, Jaiswal et al characterized the ARE in the hNQO1 gene in a hepatoma cell line and suggested that multiple AP-1 proteins bind to this DNA sequence (Li and Jaiswal, 1992, 1994). In this report, we have performed functional assays to identify the *cis* DNA elements important in the transcriptional regulation of NQO1 in human lung tumour cells. Our data indicate that the ARE is important in regulating NQO1 promoter activity in NSCLC. We have also examined binding activity of Fos and Jun protein family members to the hNQO1 ARE and demonstrate Fra1, Fra2 and JunB binding activity only in NSCLC cell lines that constituitively express NQO1.

*Received 20 April 1998*
*Revised 6 August 1998*
*Accepted 7 August 1998*

*Correspondence to:* D Ross

### MATERIALS AND METHODS

### Cell lines and cell culture

The human NSCLC cell lines (NCI-H460, NCI-H520, A549) were obtained from the American Type Culture Collection (ATCC, Rockville, MD, USA) and were maintained in modified Eagles medium (MEM) supplemented with 2 mM L-glutamine, 100 units $ml^{-1}$ penicillin, 100 μg $ml^{-1}$ streptomycin and 10% fetal bovine serum (Summit Biotechnology, Fort Collins, CO, USA). The human SCLC cell lines (NCI-H146, NCI-H82, NCI-H187) were also obtained from ATCC and were maintained in RPMI-1640 with the same supplements. PCR-RFLP (polymerase chain reaction-restriction fragment length polymorphism) (Traver et al, 1997) was performed on these six cell lines to ensure wild-type or heterozygous NQO1 polymorphism status (wild-type: H460, A549, H146, H82; heterozygous: H520, H187).

### Construction of plasmids

The reporter plasmid vector, $pGL_3$ (Promega, Madison, WI, USA) contains a synthetic polyadenosine (poly-A) signal placed upstream of the firefly luciferase gene and a SV40 late poly-A signal downsteam of the reporter gene. A 1765 bp fragment (–1539 to + 115) of the hNQO1 gene was isolated from a human placental cosmid library (gift from Dr NW Gibson). To generate 5′ deletion constructs, convenient restriction enzyme sites were used to subclone the DNA fragments into the Sma I/Nco I site in $pGL_3$.

### Transient DNA transfections

Lipofection of plasmid DNA into the various human lung cells was performed as described (BRL, Gaithersburg, MD, USA). Briefly, each 35 mm plate received $1 \times 10^6$ cells, 1 µg of test plasmid, 1 ug of pCMVβgal (Clontech, Palo Alto, CA, USA) and 3 µl or 4 µl (NSCLC or SCLC respectively) of Lipofectamine (BRL, Gaithersburg, MD, USA). Cells were harvested after 24 h, lysed, and cell debris sedimented. For normalization of transfection efficiency, cells were transfected in parallel with the control plasmid, $pSV40GL_3$ (Promega, Madison, WI, USA). Cell extracts were then assayed for luciferase and β-galactosidase activities (Promega, Madison, WI, USA).

### Nuclear extracts

High salt nuclear extracts were prepared using a modification of the method of Dignam et al (1983). Briefly, washed pelleted cells ($5–10 \times 10^8$) were suspended in two packed volumes of 10 mM Hepes, pH 7.9, 1.5 mM magnesium chloride, 60 mM potassium chloride and 0.5 mM DTT, and lysed by 15 strokes of an all glass Dounce homogenizer (B type pestle). The homogenate was centrifuged to pellet the nuclei. The crude nuclei were resuspended in 0.6 ml of 20 mM Hepes, pH 7.9, 25% glycerol, 500 mM potassium chloride, 0.5 mM DTT, 1.5 mM magnesium chloride, 0.2 mM EDTA, pH 8.0 and 0.1 mM EGTA, pH 8.0 and gently shaken for 30 min. After centrifugation, the supernatant was dialysed for 3 h and then frozen in aliquots at –70°C. The protein concentration in the nuclear extracts were determined using the Bradford protein assay (Bio Rad, Richmond, CA, USA).

### Electrophoretic gel mobility shift assays

The double stranded oligomer used in the gel shift studies was synthesized as a complementary pair on an automated DNA synthesizer. The oligomer was end-labelled using $T_4$ polynucleotide kinase (BRL) and [32]γ-ATP (NEN, 3000 Ci $mmol^{-1}$) to a specific activity of 10–30 000 cpm and applied to a G-10 Sephadex column (Clontech, Palo Alto, CA, USA). The human NQO1 promoter fragments were the sense strand, 5′-AAATCGCAGTCACAGTGACTCAGCAGAATCTGAGCCTAGG-3′ annealed to the antisense strand, 5-′CCTAGGCTCAGATTCTGCTGAGTCACTGTGACTGCGATTT-3′ and represented sequences from –477 to – 438 and contained the ARE; the sense strand, 5′-GATTACAGGCGTGAGCACCG-3′ annealed to the antisense strand 5′-CGGTGCTCACGCCTGTAATC-3′ and represented sequences from –749 to –730 and contained the XRE; the sense strand, 5′-AACAAATTCGTCTCCACGGAGCATGTCT-3′ annealed to the antisense strand 5′-AGACATGCTCCGTGGAGACGAATTTGTT-3′ and represented mid-promoter sequences (MPR) from –527 to –499. The sense strand, 5′-tcgaGGTAATTTGCATTTCTAA-3′ was annealed to the antisense strand 5′-tcgaTTAGAAATGCAAATTACC-3′ and represented sequences –537 to –554 plus TCGA overhangs and contained the octamer (Oct-1) motif region of the mouse immunoglobin enhancer (Ephrussi et al, 1985). The sense strand, 5′-tcgaCTAGTGATGAGTCAGCCGGATC-3′ was annealed to the antisense strand 5′-tcgaGATCCGGCTGACTCATCACTAG-3′ and represented an artificial consensus AP-1 motif region (Shy et al, 1996) plus TCGA overhangs. Briefly, 3 µg of nuclear extract were incubated at 25°C for 30 min in binding buffer consisting of 20 mM Hepes, pH 7.9, 2.5 mM magnesium chloride, 1 mM DTT, 2 µg bovine serum albumin and 10% glycerol with 150 ng poly dIdC. Approximately 20 000 cpm of end-labelled DNA probe was added to the reaction and binding carried out at 25°C for an additional 30 min. The specific polyclonal antibodies were added to some of the samples after the addition of labelled DNA fragments and incubated for an additional 30 min at 25°C. Unlabelled fragments were added to some of the samples and pre-incubated for 30 min at 25°C prior to the addition of labelled fragments. Samples (20 µl) were subjected to electrophoresis on a 5% non-denaturing polyacrylamide gel (0.25× TBE) at 25°C and 20 mA for 1 h. Gels were dried and exposed to Hyperfilm (Amersham, Arlington Heights, IL, USA) at –70°C.

**Table 1** DT-diaphorase activity in cell lines from lung tumours

| Human lung cell lines | DT-diaphorase activity (nmoles $min^{-1}$ $mg^{-1}$ protein) |
|---|---|
| NSCLC/NCI-H460 | 1782 ± 72 |
| NSCLC/A549 | 2484 ± 52 |
| NSCLC/NCI-H520 | 1122 ± 56 |
| SCLC/NCI-H146 | 6 ± 0.6 |
| SCLC/NCI-H82 | 3 ± 1.4 |
| SCLC/NCI-H187 | 3 ± 1.0 |

Enzyme activities (mean ± s.d.) were determined as described (Malkinson et al, 1992).

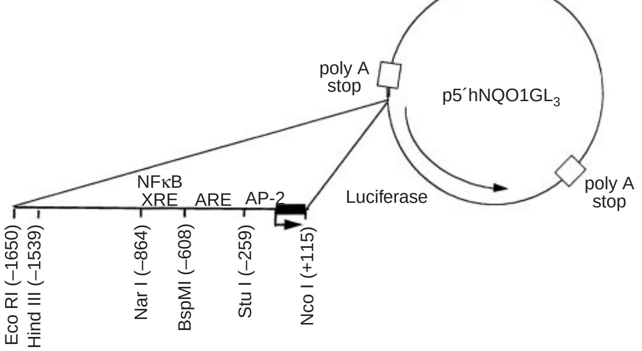

**Figure 1** Human NQO1 promoter constructs. The 5′ deletion constructs were prepared using convenient restriction enzyme sites to examine areas important for basal promoter activity. The 3′ extent of all inserts was to the Nco I site at + 115. DNA fragments were inserted into the Sma I-Nco I site in $pGL_3$

*© Cancer Research Campaign 1999*

### RESULTS

### DT-diaphorase activity in cell lines derived from human lung tumours

The six representative cell lines listed in Table 1 have well characterized morphological and growth characteristics. The NSCLC cell lines were derived from large cell (H460) squamous (H520) and adenocarcinoma (A549) histological types and each had markedly elevated DT-diaphorase activity relative to the SCLC cells (H146, H82 and H187). There was also a good correlation between DT-diaphorase activity and hNQO1 mRNA content (Malkinson et al, 1992).

### NSCLC exhibit greater hNQO1 promoter activity than SCLC

To identify and characterize potential regulatory regions within the hNQO1 promoter region, we constructed a series of hNQO1 promoter–LUC constructs with progressive deletions of the proximal and distal hNQO1 promoter starting from nucleotide –1539 (Figure 1). These constructs were transfected using lipofection into both NSCLC (see Figure 2A for H460 results) and SCLC (see Figure 2B using H146) cell lines and lysates were assayed for luciferase expression. The results of multiple experiments, summarized in Figure 2C, demonstrate different patterns of promoter activity between NSCLC and SCLC cell lines.

The largest hNQO1 construct (1539/+115) exhibits robust luciferase expression in the H460, A549, and H520 cell lines (Figure 2C). Deletion from –1539 to –864 resulted in no significant change in luciferase activity and truncation to –608, removing the NFκB and XRE elements (see Figure 1), maintained this robust activity (Figure 2C). Further deletion to –259 decreased luciferase activity by 85–90%, to a range between fivefold (H460, H520) and 23-fold (H549) over the promoterless vector. Interestingly, this region between –608 and –259 contains the *cis* element, ARE, within which lies a perfect AP-1 flanked by two AP-1-like motifs. In contrast, in the H146, H82 and H187 cell lines, the hNQO1 promoter is much less transcriptionally active (Figure 2B, C). The data showed that deletion of the region containing the AP-1 site did not substantially affect the level of luciferase expression.

### AP-1 is a component of ARE element-binding activity in lung cancer cells

To identify which proteins are bound to the ARE element, a gel shift mobility assay was employed to detect ARE-binding activity

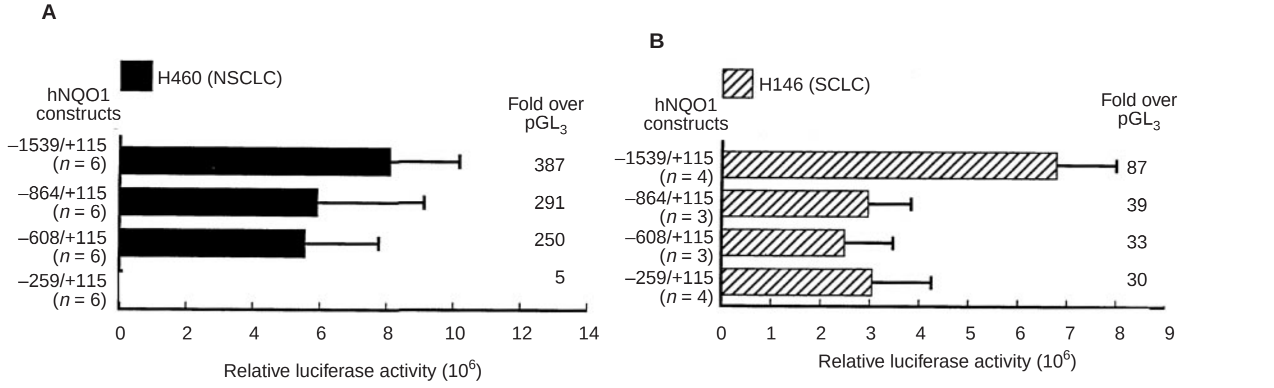

C

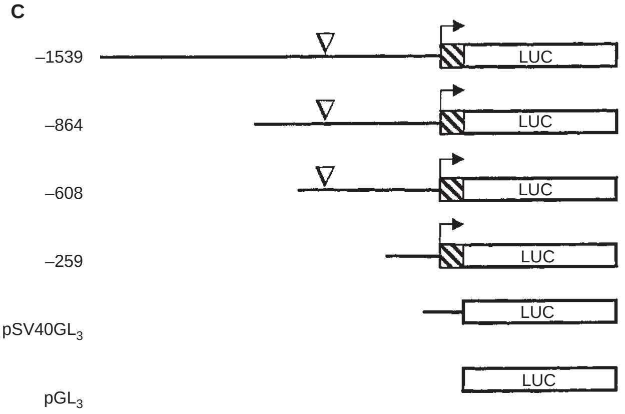

| | Relative luciferase activity | | | | | |
|---|---|---|---|---|---|---|
| | H460 | A549 | H520 | H146 | H82 | H187 |
| –1539 | 387 ± 130 (6) | 286 ± 70 (6) | 148 ± 52 (4) | 87 ± 25 (4) | 144 ± 25 (4) | 9 ± 1 (3) |
| –864 | 291 ± 177 (6) | 205 ± 30 (4) | 88 ± 22 (3) | 39 ± 14 (3) | 65 ± 23 (4) | 2 ± 1 (6) |
| –608 | 250 ± 40 (6) | 226 ± 81 (4) | 97 ± 54 (3) | 33 ± 40 (3) | 73 ± 33 (4) | 2 ± 1 (6) |
| –259 | 5 ± 1 (6) | 23 ± 4 (4) | 5 ± 1 (4) | 30 ± 7 (4) | 23 ± 4 (4) | 3 ± 2 (6) |
| pSV40GL$_3$ | 50 ± 9 (6) | 311 ± 95 (4) | 35 ± 13 (1) | 13 ± 3 (4) | 6 ± 2 (4) | 2 ± 1 (6) |
| pGL$_3$ | 1 | 1 | 1 | 1 | 1 | 1 |

**Figure 2** Basal hNQO1 promoter activity in three NSCLC and three SCLC cell lines. (**A**, **B**) Deletion constructs were prepared using convenient restriction sites. One μg of test plasmid and 1 μg of pCMVβgal were transfected into H460 cells (**A**) or H146 cells (**B**) using lipofection. After harvesting 16–18 h after transfection, lysates were assayed for luciferase and β-galactosidase activity. Data are expressed relative to 10$^6$ light units of pSV40GL$_3$ transfected in parallel. N refers to the number of experiments performed with each construct. (**C**) The luciferase activity for each plasmid construct is determined relative to that of the promoterless vector, pGL$_3$. hNQO1 exon 1 is shown as a stippled box; values are shown plus or minus s.e.m.; → indicates the transcription start site; ▽ indicates the position of the ARE (antioxidant response element); (*n*) indicates the number of experiments performed with each construct

© Cancer Research Campaign 1999

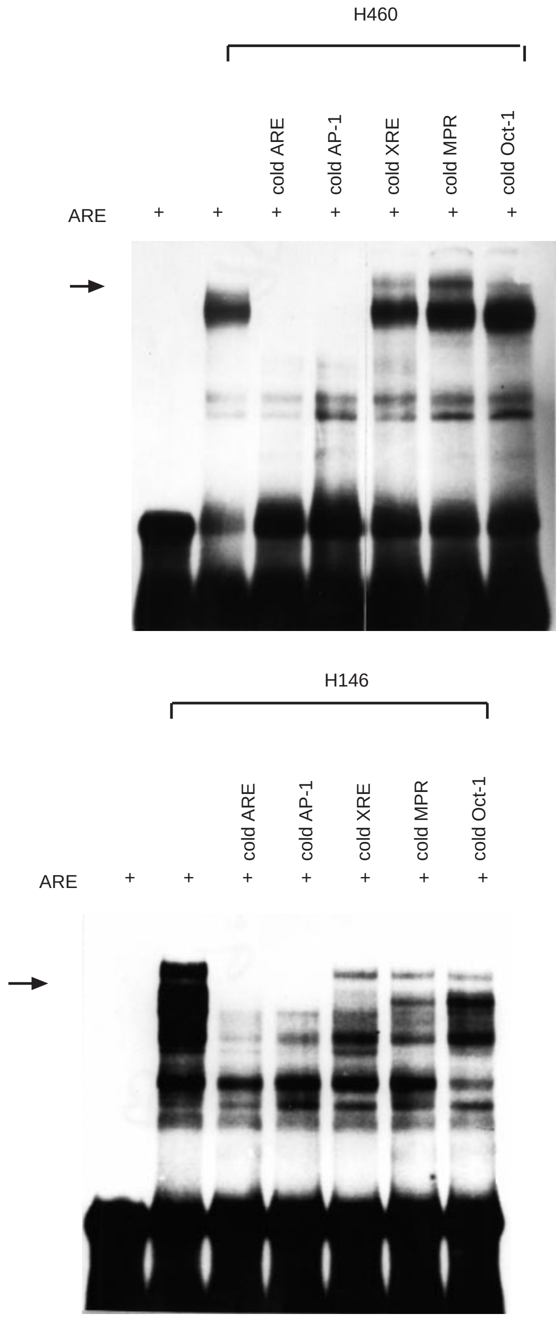

**Figure 3** Gel mobility shift assay with cold competitor oligomers. [32]P-ARE oligomer was incubated with nuclear extracts from either H460 NSCLC (top panel) or H146 SCLC (bottom panel) in the presence of either 0 or 50-fold molar excess of cold ARE, AP-1, XRE, MPR or Oct-1. The incubations were then electrophoresed on nondenaturing gels. The arrow indicates the area of major binding activity

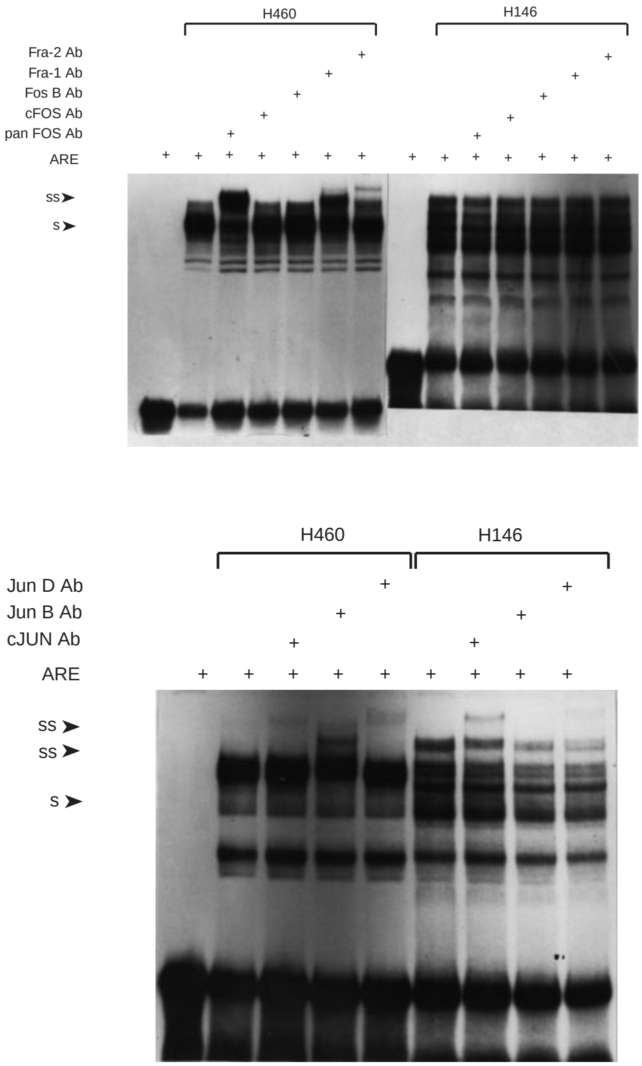

**Figure 4** Gel mobility shift assay with various specific AP-1 antibodies. [32]P-ARE oligomer was incubated with nuclear extracts from either H460 NSCLC (left panel) or H146 SCLC (right panel) followed by the addition of specific antibodies as indicated. The positions of the DNA–protein shifted complexes (s) and the supershifted complexes (ss) are indicated by the arrows in nuclear extracts prepared from both NSCLC and SCLC with a [32]P-labelled synthetic oligonucleotide spanning –477 to –438 of the hNQO1 promoter. In nuclear extracts prepared from two representative cell lines, H460 and H146, we detected specific ARE-binding activity. This binding activity was abolished by the presence of a 50-fold excess of either unlabelled ARE or unlabelled AP-1 consensus oligomer, suggesting that the same binding proteins are likely to interact with both ARE and AP-1 elements. In contrast, the unlabelled oligomers XRE, MPR, and Oct-1 were unable to compete any of the specific complexes (Figure 3). Nuclear extracts prepared from the remaining four cell lines gave similar results (data not shown).

### Differential binding of AP-1 proteins to the hNQO1 ARE fragment

To characterize further the protein complex binding to the AP-1-binding site within the ARE region of the hNQO1 gene, nuclear extracts from NSCLC or SCLC were incubated with antibodies against panFos, cFos, FosB, Fra1, Fra2 (Figure 4, top panel), and cJun, JunB, JunD (Figure 4, bottom panel) prior to detection of

 *© Cancer Research Campaign 1999*

DNA–protein interactions by gel mobility shift assay. The antibodies against panFos, Fra1, Fra2, cJun, JunB and JunD induced a supershift of the labelled DNA probe incubated with nuclear extracts from H460 (Figure 4), A549 and H520 (data not shown) lung cells, indicating that their respective antigens participate in the formation of the complex bound to this *cis* element. In H146 nuclear extracts (Figure 4; H82 and H187 data not shown), however, only antibodies against cJun and JunD induced a supershift with this same labelled DNA probe. Antibodies to cFos and FosB did not produce supershifts in H460 (Figure 4, left panel). Supershifts using pan Fos, cFos, FosB, Fra1, Fra2, and JunB were not observed using SCLC nuclear extracts (Figure 4). All gel shift experiments were performed multiple times (at least five) and produced similar results.

### DISCUSSION

Although many studies of hNQO1 regulation have been reported, these have been predominantly in liver systems (Jaiswal et al, 1988; Li and Jaiswal, 1992). The molecular mechanisms underlying increased NQO1 gene expression and activity in human NSCLC are unknown. To examine NSCLC-specific expression of NQO1, we transiently transfected a panel of deletion constructs containing the hNQO1 promoter and the luciferase reporter gene into both NSCLC and SCLC human cell lines. Deletion analysis of the largest hNQO1 promoter region demonstrated that a 359 bp proximal region (–608 to –259), which contains the ARE, was critical in mediating basal expression in NSCLC. Deletion of the sites for NFκB and AP-2 and the XRE did not significantly affect hNQO1 promoter activity in either the NSCLC or SCLC cell lines.

Gel mobility shift experiments demonstrated the specific binding of nuclear proteins to an oligomer containing the human NQO1 ARE. Competition for binding to radiolabelled ARE was observed with an oligomer containing an AP-1 site, but not with oligomers containing the human NQO1 XRE, MPR or Oct-1 oligomers. Antibody supershift experiments using the broad-spectrum anti-Fos (antibody that reacts with all known Fos family members) strongly suggest the participation of Fos family proteins in the formation of the complexes that bind the ARE only in nuclear extracts from NSCLC. A more detailed supershift analysis using antibodies specific for each family member suggests that Fra1, Fra2 and JunB are the AP-1 members found in NSCLC but not in SCLC.

As early response genes, the Jun/Fos family is tightly regulated during cellular differentiation (Karin et al, 1997) and rapidly induced following growth factor stimulation (Coussens et al, 1994) or in proliferation processes (Angel and Karin, 1991). AP-1 activity has been associated with the regulation of many genes (Busslinger and Bergers, 1994) including the positive regulation of many phase II drug metabolizing genes in vitro (Diccianni et al, 1992; Friling et al, 1992; Prestera et al, 1993).

The patterns of expression of individual Fos family members overlap but are not identical (Cohen and Curran, 1988; Angel and Karin, 1991). Fra1 and Fra2 are both expressed at significant levels in cycling cells, in contrast to cFos and FosB (Kovary and Bravo, 1992). Fra1 has been identified in keratinocytes (Welter et al, 1995) and cardiac tissue (Milivojevic and Gardner, 1995). Fra1 is a unique member of the Fos gene family which is also under positive control by AP-1 activity. It is capable of binding different Jun proteins in a manner similar to that of other members of the Fos family (Ryseck and Bravo, 1991). Unlike cFos, Fra1 lacks a transactivation domain (Suzuki et al, 1991; Wisdom and Verma, 1993) yet possesses oncogenic potential (Bergers et al, 1995). Even though Fra1 lacks a transactivation domain, it could, in theory, limit the Jun protein pool by competing for dimerization partners. Fra1 has recently been shown to be a negative regulator of antioxidant-mediated AP-1 activity in HeLa cells (Yoshioka et al, 1995) and hNQO1 promoter activity in HepG2 human liver cells (Venugopal and Jaiswal, 1996). However, the effects of Fra1 can also be dependent on cellular content. Expression studies with the human atrial natriuretic factor (ANF) gene revealed divergent regulation by Fra1 in rat cardiocytes (Milivojevic and Gardner, 1992). Interestingly, overexpressing Fra1 in atrial myocytes mimicked the suppressant effects of cFos, while in the ventrical myocyte, Fra1 behaved as a typical transcriptional activator of ANF promoter activity (Milivojevic and Gardner, 1995). As to JunB, the presence of JunB-binding activity in NSCLC cell lines is in agreement with its possible role in NQO1 expression since JunB is an efficient activator of promoters containing multimerized AP-1 sites (Chiu et al, 1989). Only the ARE in the human NQO1 gene contains a centrally-located perfect AP-1 site flanked by two imperfect AP-1 elements (Jaiswal et al, 1988). Additional studies to confirm AP-1's role in positive regulation of hNQO1 expression in lung cancer cells, will require co-transfection experiments in SCLC with vectors expressing the various absent AP-1 members. Given the potential for temporal differences in the activation of the individual AP-1 members and the stabilities of their protein products, such diversity of expression may represent novel mechanisms controlling the transcription of hNQO1 during lung cancer pathogenesis.

In summary, our results indicate that the ARE is critical to basal NQO1 expression in lung tumours (NSCLC) expressing high levels of DT-diaphorase. This initial investigation shows that Fra1, Fra2 and JunB binding activities are not detected in SCLC and thus suggests that SCLC lack the *trans*-activating factors of the AP-1 family that mediate differential NQO1 expression in lung tissue.

### ACKNOWLEDGEMENTS

This work was supported by grant CA51210 from the National Institute of Health.

### REFERENCES

Angel P and Karin M (1991) The role of Jun, Fos and the AP-1 complex in cell proliferation and transformation. *Biochim Biophys Acta* **1072**:129–157

Benson AM, Hunkeler MJ and Talalay P (1980) Increase of NAD(P)H: quinone reductase by dietary antioxidants. Possible role in protection against carcinogenesis and toxicity. *Proc Natl Acad Sci USA* **77**: 5216–5220

Berger MS, Talcott RE, Rosenblum ML, Silva M, Ali-Osman F and Smith M (1985) The use of quinones in brain tumor chemotherapy. Preliminary results from preclinical investigations. *J Toxicol Environ Health* **16**: 713–719

Bergers G, Graninger P, Braselmann S, Wrighton C and Bussinger M (1995) Transcriptional activation of the Fra-1 gene by AP-1 is mediated by regulatory sequences in the first intron. *Mol Cell Biol* **15**: 3748–3758

Beyer RE, Segura-Aguilar J, Lind C and Castro VM (1987) DT-diaphorase activity in various cells in culture with emphasis on induction in ascites hepatoma cells. *Chemica Scripta* **27A**: 145–150

Busslinger M and Bergers G (1994) Identification of AP-1 regulated genes. In *The Fos and Jun Families of Transcription Factors*, Angel PE and Herrlich PA (eds), pp. 133–150. CRC Press: Boca Raton, FL

© Cancer Research Campaign 1999 

Chiu R, Angel P and Karin M (1989) JunB differs in its biological properties from, and is a negative regulator of cJun. *Cell* **59**: 979–986
Cohen DR and Curran T (1988) Fra-1: a serum-inducible cellular immediate: early gene that encodes a fos-related antigen. *Mol Cell Biol* **8**: 2063–2069
Coussens LM, Yokoyama K and Chiu R (1994) Transforming growth factor beta 1-mediated induction of JunB is selectively inhibited by expression of Ad. 12-E1a. *J Cell Physiol* **160**: 435–444
DeLong MJ, Prochaska HJ and Talalay P (1986) Induction of murine hepatoma cells by phenolic antioxidants, azo dyes and other chemoprotectors: a model system for the study of anticarcinogens. *Proc Natl Acad Sci USA* **83**: 787–791
Diccianni MB, Imagawa M and Muramatsu M (1992) The dyad palindromic glutathione transferase P enhancer binds multiple factors including AP-1. *Nucleic Acids Res* **20**: 5153–5158
Dignam JD, Lebovitz R and Roeder RG (1983) Accurate transcription initiation by RNA polymerase II in a soluble extract from isolated mammalian nuclei. *Nucleic Acids Res* **11**: 1475–1489
Ephrussi A, Church A, Tonegawa S and Gilbert W (1985) β lineage-specific interactions of an immunoglobulin enhancer with cellular factors in vivo. *Science* **227**: 134–140
Friling RS, Bergelson S and Daniel V (1992) Two adjacent AP-1 like binding sites form the electrophilic-responsive element of the murine glutathione S-transferase Ya subunit gene. *Proc Natl Acad Sci USA* **89**: 668–672
Jaiswal AK (1991) Human NAD(P)H:quinone oxidoreductase (NQO1) gene structure and induction by dioxin. *Biochemistry* **30**: 10647–10653
Jaiswal AK, McBride OW, Adesnik M and Nebert DW (1988) Human dioxin inducible cytosolic NAD(P)H:menadione oxidoreductase. *J Biol Chem* **263**: 13572–13578
Joseph P, Jaiswal AK, Stobbe CC and Chapman JD (1994) The role of specific reductases in the intracellular activation and binding of 2-nitroimidazoles. *Int J Radiat Oncol Biol Phys* **29**: 351–355
Karin M, Liu Z and Zandi E (1997) AP-1 function and regulation. *Curr Opin Cell Biol* **9**: 240–246
Kovary K and Bravo R (1992) Existence of different Fos/Jun complexes during the $G_0$ to $G_1$ transition and during exponential growth in mouse fibroblasts: differential role of Fos proteins. *Mol Cell Biol* **12**: 5015–5023
Li Y and Jaiswal AK (1992) Regulation of human NAD(P)H:quinone reductase gene. Role of AP-1 binding site contained within human antioxidant response element. *J Biol Chem* **267**: 15097–15104
Li Y and Jaiswal AK (1994) Human antioxidant response element regulation of type 1 NAD(P)H:quinone oxidoreductase gene expression. *Eur J Biochem* **226**: 31–39
Malkinson AM, Siegel D, Forrest GL, Gazdar AF, Oie HK, Chan DC, Bunn PA, Mabry M, Dykes DJ, Harrison SD Jr and Ross D (1992) Elevated DT-diaphorase activity and messenger RNA content in human non-small cell lung carcinoma: relationship to the response of lung tumour xenografts to mitomycin C. *Cancer Res* **52**: 4752–4757
Milivojevic BK and Gardner DG (1992) Divergent regulation of the human atrial natriuretic peptide gene by c-jun and c-fos. *Mol Cell Biol* **12**: 292–301
Milivojevic BK and Gardner DG (1995) Fra-1, a Fos gene family member that activates atrial natriuretic peptide gene transcription. *Hypertension* **25**: 679–682
Prestera T, Holtzclaw WD, Zhang Y and Talalay P (1993) Chemical and molecular regulation of enzymes that detoxify carcinogens. *Proc Natl Acad Sci USA* **90**: 2965–2969
Ryseck RP and Bravo R (1991) cJun, Jun B, and Jun D differ in their binding affinities to AP-1 and CRE consensus sequences: effect of Fos proteins. *Oncogene* **6**: 533–542
Schlager JJ and Powis G (1990) Cystolic NAD(P)H: (quinone acceptor) oxidoreductase in human normal and tumor tissue: effects of cigarette smoking and alcohol. *Int J Cancer* **45**: 403–409
Shy ME, Shi Y, Wrabetz L, Kamholz J. and Scherer SS (1996) Axon–schwann cell interactions regulate the expression of c-jun in Schwann cells. *J Neurosci Res* **43**: 511–525
Smitskamp-Wilms E, Giaccone G, Pinedo HM, Van der Laan BFAM and Peters GJ (1995) DT-diaphorase activity in normal and neoplastic human tissues: an indicator for sensitivity to bioreductive agents. *Br J Cancer* **72**: 917–921
Suzuki T, Okuno H, Yoshida T, Endo T, Nishina H and Iba H (1991) Differences in transcriptional regulatory function between cFos and Fra-2. *Nucleic Acids Res* **19**: 5537–5542
Szabo E, Riffe ME, Steinberg SM, Birrer MJ and Linnoila RL (1996) Altered cJun expression: an early event in human lung carcinogenesis. *Cancer Res* **56**: 305–315
Talalay P and Prochaska HJ (1987) Mechanisms of induction of NAD(P)H:quinone reductase. *Chemica Scripta* **27A**: 61–66
Talalay P, DeLong MJ and Prochaska HJ (1988) Identification of a common chemical signal regulating the induction of enzymes that protect against chemical carcinogenesis. *Proc Natl Acad Sci USA* **85**: 8261–8265
Traver RD, Siegel D, Beall HD, Phillips RM, Gibson NW, Franklin WA and Ross D (1997) Characterization of a polymorphism in NADP(H):quinone oxidoreductase (DT-diaphorase). *Br J Cancer* **75**: 69–75
Venugopal R and Jaiswal AK (1996) Nrf1 and Nrf2 positively and cFos and Fra-1 negatively regulate the human antioxidant response element-mediated expression of NAD(P)H:quinone reductase $_1$gene. *Proc Natl Acad Sci USA* **93**: 14960–14965
Wang B and Williamson G (1994) Detection of a nuclear protein which binds specifically to the antioxidant responsive element (ARE) of the human NAD(P)H:quinone oxidoreductase gene. *Biochim Biophys Acta* **1219**: 645–652
Welter JF, Crish JF, Agarwal C and Eckert RL (1995) Fos-related antigen (Fra-1), JunB and JunD activate human involucrin promoter transcription by binding to proximal and distal AP-1 sites to mediate phorbal ester effect on promoter activity. *J Biol Chem* **270**: 12614–12622
Wisdom R and Verma IM (1993) Transformation by Fos proteins requires a C-terminal transactivation domain. *Mol Cell Biol* **13**: 7429–7438
Xanthoudakis S, Miao G, Wang F, Pan YC and Curran T (1992) Redox activation of Fos-Jun DNA binding activity is mediated by a DNA repair enzyme. *EMBO J* **11**: 3323–3335
Yoshioka K, Deng T, Cavigelli M and Karin M (1995) Antitumor promotion by phenolic antioxidants: inhibition of AP-1 activity through induction of Fra expression. *Proc Natl Acad Sci USA* **92**: 4972–4976

*© Cancer Research Campaign 1999*
